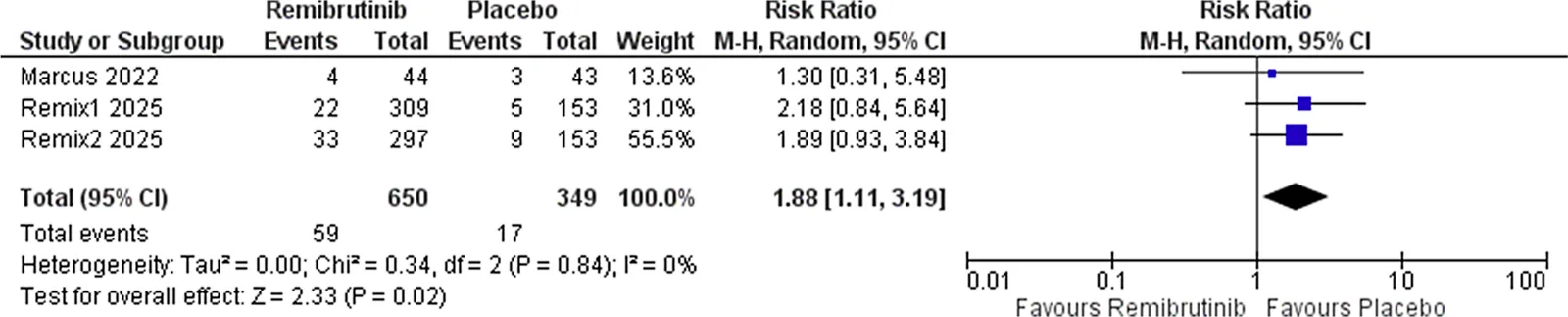# Erratum

**DOI:** 10.1159/iaa/adfag022

**Published:** 2026-09-21

In the article “Safety and Efficacy of Remibrutinib for Chronic Spontaneous Urticaria: A Systematic Review and Meta-Analysis” [*Int Arch Allergy Immunol*. 2026;187(6):635–646. https://doi.org/10.1159/000548302] by Muhammad Burhan et al., online supplementary Figure 11 has been corrected.

Following publication, the authors reviewed their data and found that an error occurred during the compilation of their forest plot of online supplementary Figure 11. This error arose from an internal data entry oversight during the analysis phase.

The corrected online supplementary Figure 11 has been provided here. In addition, the corrected Results sentence in the manuscript text is as follows:

“Three studies examined nasopharyngitis cases in both groups. The pooled analysis, as depicted in online supplementary Figure 11, demonstrated a statistically significant increase in the risk of nasopharyngitis in the remibrutinib group compared with placebo (RR: 1.88, 95% CI: 1.11–3.19, *I*² = 0%, *p* = 0.02).”

**Figure:**